# Synergistic Properties of Methylxanthine with Cadmium and Mercury on Dermatophytes

**DOI:** 10.4103/0974-777X.62878

**Published:** 2010

**Authors:** Ali Abdul Hussein S. AL-Janabi

**Affiliations:** *Department of Clinical Laboratory, College of Pharmacy, University of Kerbala, Iraq*

**Keywords:** Cadmium, Dermatophytes, Mercury, Methylxanthinel

## Abstract

**Background:**

Methylxanthine has many antimicrobial effects on different types of organisms. Synergism actions between methylxanthine and cadmium or mercury on dermatophytes were tested.

**Aims:**

To determine the synergistic property between methylxanthine and cadmium or mercury on the growth of dermatophytes.

**Materials and Methods:**

Two isolated strains of dermatophytes (*Trichophyton mentagrophytes* and *Epidermophyton floccosum*) that clinical isolated were treated with the mixtures of methylxanthine either with cadmium or with mercury by using colony diameter method. Percentage inhibition was also calculated based on perpendicular colony diameters of grown fungi.

**Results:**

Cadmium had high ability to increase inhibitory action of methylxanthine agents on dermatophytes. *Epidermophyton floccosum* revealed more susceptibility to tested mixture than those of *Trichophyton mentagrophytes*. Mercury exhibited variable effects on dermatophytes after mixing with methylxanthine.

**Conclusions:**

Effective concentrations of methylxanthine on dermatophytes decreased in the presence of cadmium and mercury. Variable susceptibility of two species of dermatophytes to prepared mixture was noted.

## INTRODUCTION

Dermatophytes consider very important pathogenic fungi for human. They cause common skin disease known as dermatophytosis or tinea.[1] In present time, many pharmaceutical drugs are usually described for treatment of dermatophytosis. Most of them need a long periods of time with a failure in some cases.

Methylxanthine that consists of caffeine, theophylline and theobromine are found in many species of plants with various concentrations. *Coffea arbacia*, *Cola nitida*, *Camellia sinensis* are main natural source of these compounds.[2] Antifungal activity of methylxanthine when they presence in pure state or in their producing plants was reported. Infusion type of tea that produced from black and green tea was showed highest activity on *Penicillium.*[3] Caffeine as one components of tea exhibited fungicidal action on four fish pathogenic species of Saprolegniaceae.[4] Furthermore, Jayaratna *et al*.[5] found that colony diameter of fungus *Monacrosporium ambrosium* grown on caffeine containing media was significantly less than on the control media. Caffeine can effect also on fungal products as noted with preventing aflatoxin production by *Aspergillus flavus*.[6]

Microelements, such as cadmium and mercury are necessary for fungi development through incorporating them within the internal structures of mycelium. Each kilogram of fungal body found to contain 0.2 to 130 mg of cadmium and 0.02 to 64 mg of mercury.[7] Otherwise, some types of fungi are sensitive to cadmium, while others are not. Resistance to cadmium may be resulted from either the ability of fungi to metabolize it[8] or accumulation of cadmium in their vacuolar compartments.[9] For many species of fungi, cadmium may act as toxic agent. It inhibit the growth of *Paecilomyces farinosus* at concentration of 300 ppm,[10] while five of ten species of fungi were inhibited in soil supplemented with 1000 μg of cadmium per gram.[11]

According to spectroscopic results, Nafisi *et al*.[12] found that cadmium and mercury bind strongly to caffeine and theophylline. Thus, investigation for any possible correlation between activities of methylxanthine compounds when they mixing with cadmium or mercury on dermatophytes was the aim of this study.

## MATERIALS AND METHODS

### Organisms

Two strains of dermatophytes were clinical isolated from old male patient (26 years) infected with dermatophytosis at AL-Hussein general hospital of Kerbala province in February 2009. Skin scales of fungal lesion were cultured on Sabouraud's glucose agar of the following components; glucose 20 g, peptone 10 g, agar 15 g, chloramphenicol 0.05 g and 1000 ml of distilled water. Cultures were incubated at 28°C for two weeks. Grown fungi were diagnosed according to criteria recorded by Rippon[13] and Emmons.[14] The isolated strains were: *Trichophyton mentagrophytes* and *Epidermophyton floccosum*.

### Chemical agents

Theophylline, caffeine, and theobromine were purchased from HiMedia, Mumbai-India. Cadmium sulphate and mercury (1) nitrate were purchased from (BDH, Poole-England). Clotrimazole was purchased from Arabic drug industry (ADI)-Iraq. Selective concentration of methylxanthine that mixed with heavy metals based on primary experimented was as following: 0.5 mg/ml of caffeine, 1.5 mg/ml of theophylline and 5 mg/ml of theobromine.

### Antifungal assay

Colony diameter method recorded by Kücüc and Kivan[15] was used. Various concentrations of unique or mixed compounds including 50, 12.5, 6.25, 3.125 and 1.56 μg/L were mingled with melting prepared Sabouraud's glucose agar. Then, poured in sterile Petri dishes. A disk (9 mm) of old grown fungi (at 28°C for 1 week) was inoculated on the center of culture media. Plates were incubated at 28°C for 1 week. Two controls were used, including clotrimazole (200 μg/ml) as positive control and untreated media as negative control. Each experiment was repeated three times with triplicates of each concentration. Perpendicular colony diameters (mm) of grown strains were measured and percentage inhibition calculated according to the formula: $\text{Percentage inhibition}=\frac{\left(\text{C−T}\right)\times 100}{\text{C}}$

Where, C = colony diameter (mm) of the control

T = colony diameter (mm) of the test plate

### Determination of minimal inhibitory concentration

Minimal inhibitory concentration (MICs) were determined using microdilution method recommended by NCCLS.[16] Mixture of methylxanthine with cadmium and mercury were twofold diluted in Sabouraud's glucose broth. A 100 μl of each dilution was dispensed in well of microdilution plates (96 wells). Well was inoculated with 50 μl of medium containing fungal cells (2 × 10^4^ cfu/ml). The inoculated microdilution plates were incubated at 28°C for 72 h and examined for visible growth in order to determine MIC.

## RESULTS

**Figure 2:**
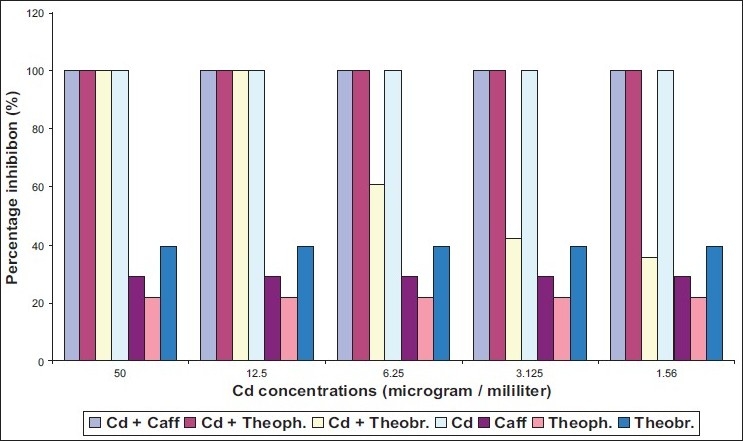
Effect of mixing cadmium (Cd) with methylxanthine on *E. floccosum*

**Figure 3:**
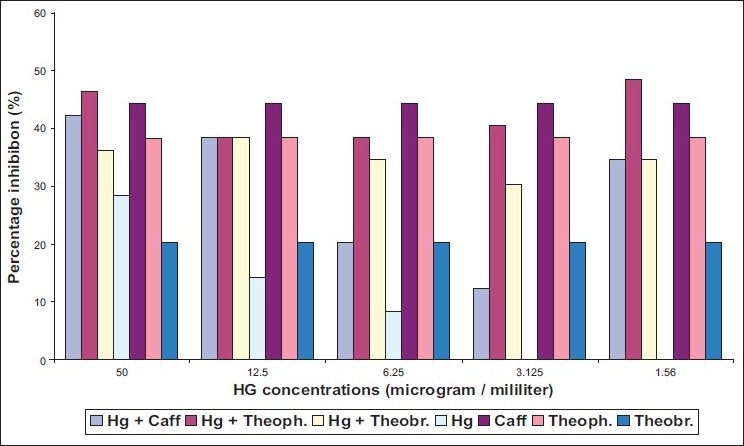
Effect of mixing mercury (Hg) with methylxanthine on *T. mentagrophytes*

**Figure 4:**
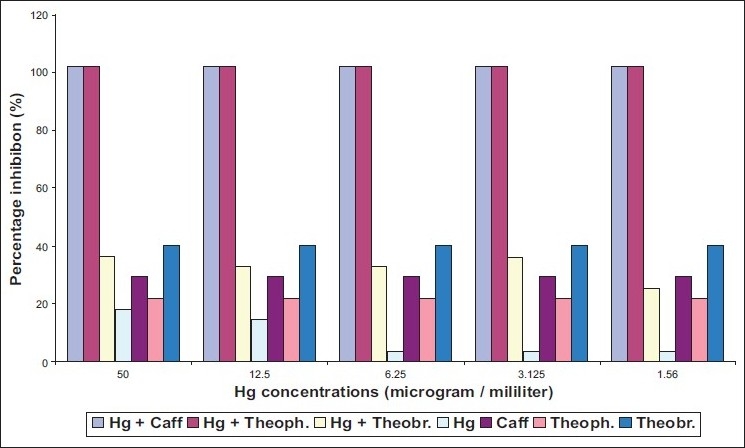
Effect of mixing mercury (Hg) with methylxanthine on *E. floccosum*

The ability of two different species of dermatophytes to grow on media containing methylxanthine with two heavy metals was determined. *Epidermophyton floccosum* found to be more susceptible to caffeine and theophylline in presence of either cadmium or mercury [Figures 2, 4]. Meanwhile, inhibitory action of theobromine on *E. floccosum* was affected by cadmium through increasing the percentage inhibition [Figure 3].

**Figure 1:**
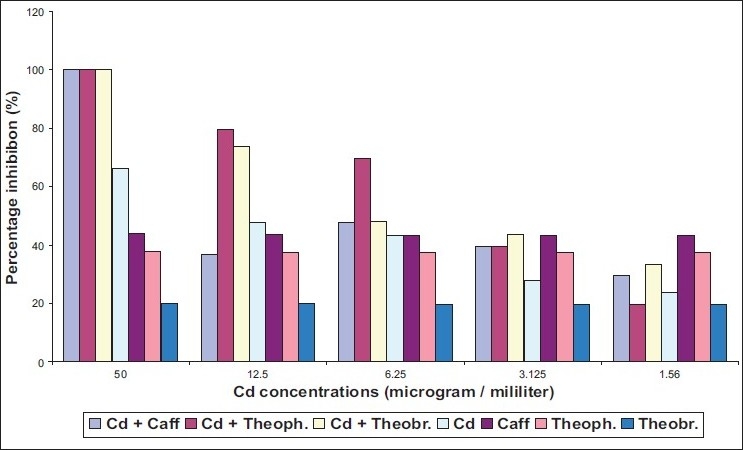
Effect of mixing cadmium (Cd) with methylxanthine on *T. mentagrophytes*

Three tested methylxanthine showed high inhibition ratio on *T. mentagrophytes* in the presence of cadmium at concentrations ranging from 12.5 μg/L to 50 μg/L [Figure 1] with MIC value for mixture of Cd with caffeine, Cd with theophylline, and Cd with theobromine equal to 40, 35, 30 respectively [Table 1]. Mercury had no effected in caffeine action on *T. mentagrophytes* [Figure 2]. By contrast, inhibitory activity of theophylline and theobromine was elevated in the presence of mercury. However, cadmium without mixing with any compound revealed more toxic effects on both strains of fungi compared with mercury [Figure 1, 3].

**Table 1:** MIC of cadmium and mercury in isolated dermatophytes after mixing with methylxanthines

Heavy metals	MIC concentrations (μg/L)	
	
	*T. mentagrophytes*	*E. floccosum*
Cd+ caffeine	40	0.39
Cd + Theophylline	35	0.39
Cd + theobromine	30	8
Hg + caffeine	>50	0.39
Hg+ theophylline	>50	0.39
Hg+ theobromine	>50	>50

Methylxanthine concentrations were: Caffeine=0.5 mg/ml, theophylline=1.5 mg/ml and theobromine=5 mg/ml

## DISCUSSION

Methylxanthines are an important group of purine alkaloids that share in xanthine structure. They have many pharmaceutical applications in human body. Thus, they used widely in medical field. Principle utilization of theophylline is for treatment of asthma, caffeine for stimulating the central nervous system, and theobromine for diuretic action.[2]

Methylxanthine, cadmium or mercury may have no effect on fungi when they present alone. The fungus *Chrysosporium keratinophilum* could be resistance to cadmium as high as 560 ppm.[17] Incorporated of cadmium into several types of low and high molecular weight proteins may assist detoxification of cadmium.[8] On the other hand, caffeine exerted a negative effect on germination, on the nuclear duplication cycle and on first septum formation of *Aspergillus nidulans*.[18]

Previously, caffeine and theophylline proved to have the ability to inhibit four species of dermatophytes that infected human skin.[19] Their inhibitory action required high concentrations to cease fungal growth. At specific concentration, heavy metals became toxic agents for many living organisms. Thus, mixing of methylxanthine compounds with trace amounts of common toxic metals was tested on dermatophytes. Cadmium and mercury were chosen to be enhancement factors for methylxanthine activity on two species of dermatophytes in reducing doses.

Nafisi *et al*.[12] observed that direct and indirect interactions of caffeine and theophylline with cadmium and mercury was through bounded with O6 and N9 atoms of caffeine and O6, N9 and N7 atoms of theophylline. The present study illustrated that cadmium decreased the effective concentrations of caffeine, theophylline and theobromine that companioned with high percentage inhibition. Thus, synergism action was an acceptable explanation for such relationship. In this case, cadmium and mercury may consider adsorption factors for methylxanthine that facilitated attachment of them with fungal cell wall. However, phosphate, carboxylate and other functional groups may consider being the sites of metals absorption in fungal biomass.[20]

Uptake of cadmium and mercury in fungi is highly species-dependent.[7] According to percentage inhibition data, *E. floccosum* was more sensitive to mixture of tested agents. This may related to limited production of conidia (only macroconidia) by *E. floccosum* compared with two types of conidia (macroconidia and microconidia) produces by *T. mentagrophytes*.[13,14] Thus, *E. floccosum* has less chance to survive in presence of toxic metals.

Although mercury ions showed less inhibitory action than cadmium on dermatophytes, mixing with methylxanthine was increased the ability of mercury to inhibit fungal growth as indicated by elevating of percentages inhibition. In the mixture, mercury may have no effect on methylxanthine activity, but reversible interaction was noted when methylxanthine increased toxic effects of mercury. However, affinity of methylxanthine to interaction with mercury may be less than with cadmium.

From our results, theobromine considers less effective member of methylxanthine on the growth of dermatophytes. Cadmium elevated antifungal activity of theobromine to reach high degree of percentage inhibition, especially against *T. mentagrophytes*. By contrast, mercury play an opposite action through decreased the inhibitory action of theobromine on isolated strains. Mercury may protect *E. floccosum* from the action of theobromine. This was confirmed by decreasing percentage inhibition compared with theophylline.

## CONCLUSIONS

The ability of methylxanthine to inhibit dermatophytes was increased in presence of cadmium or variable concentration of mercury. *E. floccosum* exhibited more susceptibility to the mixture of methylxanthine with cadmium and mercury.

## References

[1] Chong AH, Sinclair RD (1998). Superficial fungal infections. Curr Ther.

[2] Scheindlin S (2007). A new look at the xanthine alkaloids. Mol Interv.

[3] Czerwińska E, Piotrowski W, Tom Rok (2006). Microbiological Purity of Tea and its Antimicrobial Activity.

[4] Prabhuji SK, Srivastava GC, Rizvi SJ, Mathur SN (1983). 1,3,7-trimethylxanthine (caffeine); a new natural fish fungicide. Experientia.

[5] Jayaratna NB, Karunaratne DN, Kumar NS (2007). Effect of caffeine and epigallocatechin gallate (Egcg) on sporulation, spore germination and mycelial growth of Monacrosporium ambrosium, ectosymbiote of the shot-hole borer beetle of tea.

[6] Maraqa A, AL-Sharo'a NF, Farah H, Elbjeirami WM, Shakya AK, Sallal AJ (2007). Effect of Nigella sativa extract and oil on aflatoxin production by Aspergillus flavus. Turk J Biol.

[7] Lodenius M, Kuusi T, Laaksovirta K, Liukkonen-Lilija H, Piepponen S (1981). Lead, cadmium and mercury contents of fungi in Mikkeli, SE Finland. Ann Bot Fennici.

[8] Razak AA (1989). Incorporation of cadmium into proteins in a cadmium tolerate fungi. Biol Trace Elem Res.

[9] Blaudez D, Botton B, Chalot M (2000). Cadmium uptake and subcellular compartmentation in the ectomycorrhizal fungus *Paxillus involutus*. Microbiology.

[10] Ropek D, Para A (2003). The effect of heavy metal ions and their complexions upon growth, sporulation and pathogenicity of the entomopathogenic fungus *Paecilomyces farinosus*. Polish J Environ Stud.

[11] Babich H, Stotzky G (1977). Effect of cadmium on fungi and on interactions between fungi and bacteria in soil: Influence of clay minerals and pH. Appl Environ Microbiol.

[12] Nafisi S, Sadjadi AS, Zadeh SS, Damerchelli M (2003). Interation of metal ions with caffeine and theophylline: Stability and structural features. J Biomol Struct Dyn.

[13] Rippon Jhon W (1988). Medical mycology.

[14] Emmons CW, Binford CH, Utz JP (1970). Medical mycology.

[15] Kücüc G, Kivan M (2003). Isolation of Trichoderma Spp. and determination of their antifungal, biochemical and physiological features. Turk J Biol.

[16] National Committee for Clinical Laboratory Standards (2002). Reference method for broth dilution antifungal susceptibility testing of filamentous fungi. Approved standard M38-A.

[17] Kushwaha RK (2000). The genus Chrysosporium, its physiology and biotechnological potential. Revista Iberoamericana de Micología, Bilbao.

[18] Yuvamoto PD, Said S (2007). Germination, duplication cycle and septum formation are altered by caffeine, caffeic acid and cinnamic acid in *Aspergillus nidulans*. Microbiology.

[19] AL-Janabi, Ali AS (2004). Treatment of Dermatophytoses by drugs containing some purine compounds. Ph.D. Thesis. AL-Mustansiryia Univ. College of science. (Arabic).

[20] Tobin JM, Cooper DG, Neufeld RJ (1984). Uptake of metal ions by Rhizopus arrhizus biomass. Appl Environ Microbiol.

